# Supplementary material for: Genetic Screening of Targeted Region on the Chromosome 22q11.2 in Patients with Microtia and Congenital Heart Defect
Source: Genes (Basel). 2023 Apr 7;14(4):879. doi: 10.3390/genes14040879 (PMC10137977; doi:10.3390/genes14040879)
Supplement: Supplementary file 1 [file genes-14-00879-s001.zip › genes-2243642-supplementary.pdf]

Supplementary Material

Table S1 Primers and probes used for duplex qRT-PCR

CNV	Primer/probe	Sequence of primers and fluorescence labeling probes
DUP1	Forward	5′-ATCTCCTGTCAGCTCTGCGATT-3′
	Reverse	5′-AAGGAACGCATCCAACCACTAC-3′
	Probe	5′-FAM-TTGCACCTACCATAGTC-MGB-3′
DUP2	Forward	5′-AGAGGCTCACACTAGCAACAAAGAT-3′
	Reverse	5′-CAAGGCACCGTCCTGAATTATACT-3′
	Probe	5′-FAM-CACCACCCTTTGGAAG-MGB-3′
DUP3	Forward	5′-ACATCCGGCTCATCGCC-3′
	Reverse	5′-GTTGCCCCGTTAGCACATTC-3′
	Probe	5′-FAM-CCAAAGGTCCTGTGGCT-MGB-3′
DUP4	Forward	5′-CACTCCACCATCCACTCCG-3′
	Reverse	5′-TCTGAACTTTCAGGACTTGTTAGTGC-3′
	Probe	5′-FAM-CCTTTAAACCAGGAGTGC-MGB-3′
DEL1	Forward	5′-GGCAAACTTGAGCGTCTCTACA-3′
	Reverse	5′-GGCAACATTCAACCCATTACATAG-3′
	Probe	5′-FAM-ACACGTTCACCCTCC-MGB-3′
DEL2	Forward	5′-AGAAAGGAACCAAAGATCAAAAGG-3′
	Reverse	5′-CCACCTAAGACAAAGAGGATTCG-3′
	Probe	5′-FAM-CTTTCCAAGGCTTGGAC-MGB-3′

Table S2 Summary of targeted-sequencing data output

Patient	Raw Reads	Raw Base(G)	Clean Reads	Depth	Coverage
1	35883834	5.38	30802516	80.07	97.35%
2	32781824	4.92	28822130	85.38	97.73%
3	33622166	5.04	27994164	81.96	97.72%
4	34491878	5.17	28244946	80.08	98.35%
5	32460566	4.87	28562040	86.14	98.36%
6	34199606	5.13	31184198	91.31	97.74%
7	37378204	5.61	29755168	78.28	97.50%
8	35575630	5.34	29188210	81.27	98.35%
9	37089256	5.56	30159764	81.53	97.30%
10	33052802	4.96	27455932	73.65	98.19%
11	31102748	4.67	26788286	74.16	98.13%
12	37539910	5.63	33862986	94.74	98.07%
13	30253142	4.54	26569718	81.09	97.59%
14	33468350	5.02	27441592	76.49	98.28%
15	38933386	5.84	31109562	88.67	98.16%
16	26463634	3.97	23091548	66.41	97.45%
17	29144618	4.37	25305342	70.42	98.17%
18	31806286	4.77	25894938	69.22	97.50%
19	34402660	5.16	29765070	88.22	98.35%
20*	31444142	4.72	27788266	65.39	97.29%
21	33526598	5.03	29025868	80.62	97.31%
22	34205170	5.13	30492640	88.04	97.46%

* the child in the nuclear family. 21 and 22 are the father and mother in the nuclear family.

Table S3 The association between SNPs (SNP-2) and genes

Gene	SNPs	Frequency	Samples	P
CXCL10	rs368014	0.1579	2,10,12,13,15	0.0001
	rs11704009	0.0263	9	0.00009
	rs9623076	0.0526	1,6	0.00002
	rs4820360	0.0526	1,6	0.00005
DGCR2 DGCR11 DGCR12	rs738802	0.1111	2,8,14,19	0.0001
	rs396330	0.0526	7,15	0.00001

Table S4 Distribution of CNVs in samples

Sample	Counts	Length (kbp)	DUP	DUP Length (kbp)	DEL	DEL Length (kbp)	Counts (unique CNV)
1	60	763.00	43	703.60	17	59.40	22
2	60	806.70	38	699.80	22	106.90	34
3	58	733.20	37	575.70	21	157.50	25
4	57	712.20	36	583.60	21	128.60	26
5	55	809.40	38	690.30	17	119.10	27
6	59	921.70	38	775.60	21	146.10	24
7	59	956.90	37	798.70	22	158.20	24
8	11	179.70	9	167.40	2	12.30	5
9	57	828.80	35	685.30	22	143.50	21
10	56	885.90	36	765.90	20	120.00	25
11	51	753.80	34	662.70	17	91.10	22
12	58	904.90	42	792.30	16	112.60	33
13	58	855.90	33	707.50	25	148.40	27
14	52	788.40	32	649.20	20	139.20	19
15	63	894.20	42	774.70	21	106.50	28
16	48	663.80	35	539.00	13	124.80	21
17	50	952.60	37	857.00	13	95.60	25
18	62	854.10	38	730.00	24	124.10	27
19	58	646.00	40	545.20	18	100.80	25
21	50	826.30	31	688.80	19	137.50	23
22	53	797.30	39	731.00	14	66.30	24
Total	1135	16534.80	750	14123.30	385	2398.50	504
Mean	54.05	787.37	35.71	672.54	18.33	114.21	24.14

Table S5 Summary of findings

Candidate genes	functional annotation	eQTL analysis	gene-based testing	CNV analysis
SUSD2			√	√
TBX1	√		√	
DGCR8	√		√	
CXCL10		√		
CLTCL1			√	
TANGO2			√	
RIMBP3			√	
PI4KA			√	
UFD1			√	
